# Maximizing Neurovascular Outcomes of Facial Transplantation: A Comprehensive Review

**DOI:** 10.1002/ca.70067

**Published:** 2026-01-02

**Authors:** Olivia A. James, Faye Bennett

**Affiliations:** ^1^ The University of Leeds Leeds United Kingdom; ^2^ Clinical Anatomy Department The University of Leeds Leeds United Kingdom

**Keywords:** Allograft, Face transplant, Facial transplantation, Neurovascular, Plastic surgery, Reconstructive surgery

## Abstract

Facial transplantation is a division of reconstructive surgery which aims to improve the function and appearance of a face that has endured severe disfigurement. Currently, the face transplant procedure uses allogenic tissue, harvested from a brain‐dead donor, to replace damaged facial components. To date, 50 face transplants have been performed worldwide. Survival and functional success of the transplant is dependent on multiple factors; however, effective neurovascular recovery of the graft is arguably the most important. An effective vasculature system is essential to avoid necrosis and death of the transplanted tissue, and an effective nervous system is required for the graft to regain function. This comprehensive review examines and evaluates recommendations found in different studies to try and determine techniques for achieving optimal neurovascular graft outcomes. Sensory recovery of the graft occurs spontaneously through natural regeneration of the trigeminal nerve's cutaneous branches. Motor recovery, however, relies on precise coaptation of donor and recipient nerves using microsurgical sutures, reinforced by fibrin glue. Additional considerations, including parotid gland exclusion and use of a nerve graft, can further improve outcomes. Pre‐operative vascular assessment of both the donor and recipient is another crucial consideration. Computed tomography angiography, magnetic resonance angiography, and digital subtraction angiography can be used to plan and enable successful end‐to‐end or end‐to‐side anastomosis of vessels. If applied in future facial transplantation procedures, these techniques could enhance recovery, improve functional outcomes, and lead to a greater patient quality of life.

## 1 Introduction

A face transplant (FT) is a form of vascularized composite tissue allotransplantation, whereby a varying amount of tissue, with an intact circulation, is harvested from a brain‐dead donor and transplanted onto a recipient (Kantar et al. 2021). Donation after circulatory death is not routinely used due to the prolonged warm ischemic time, which increases the risk of tissue necrosis and graft failure (Morrissey and Monaco 2014; Hong et al. 2019). In general, FTs are considered life‐enhancing, rather than life‐saving procedures, with the main aims of improving facial appearance and functions such as chewing, swallowing, talking, breathing, and expression of mood (Alberti and Hoyle 2021). Though FTs are very individualized procedures, components of the graft vary and are dependent on the recipient's reconstructive needs as well as how much viable tissue is available from the donor (Barret 2014). As such, FTs can be categorized into partial or full. A ‘partial’ FT is performed if disfigurement is limited to a certain area of the face, and only a portion needs replacement (Figure 1) (Ramly et al. 2019), whereas a ‘full’ FT, replacing the entire superficial face, can be performed in cases of extensive damage (Figure 2) (Barret 2014). Deep structures, including bone and cartilage, can be included in both partial and full FTs depending on the depth of injury, and are used to restore the structural integrity of the recipient's face (Shokri et al. 2020). The surgery is considered to create a third identity, reflecting features of the recipient's and donor's faces (Azevedo et al. 2023).

**FIGURE 1:**
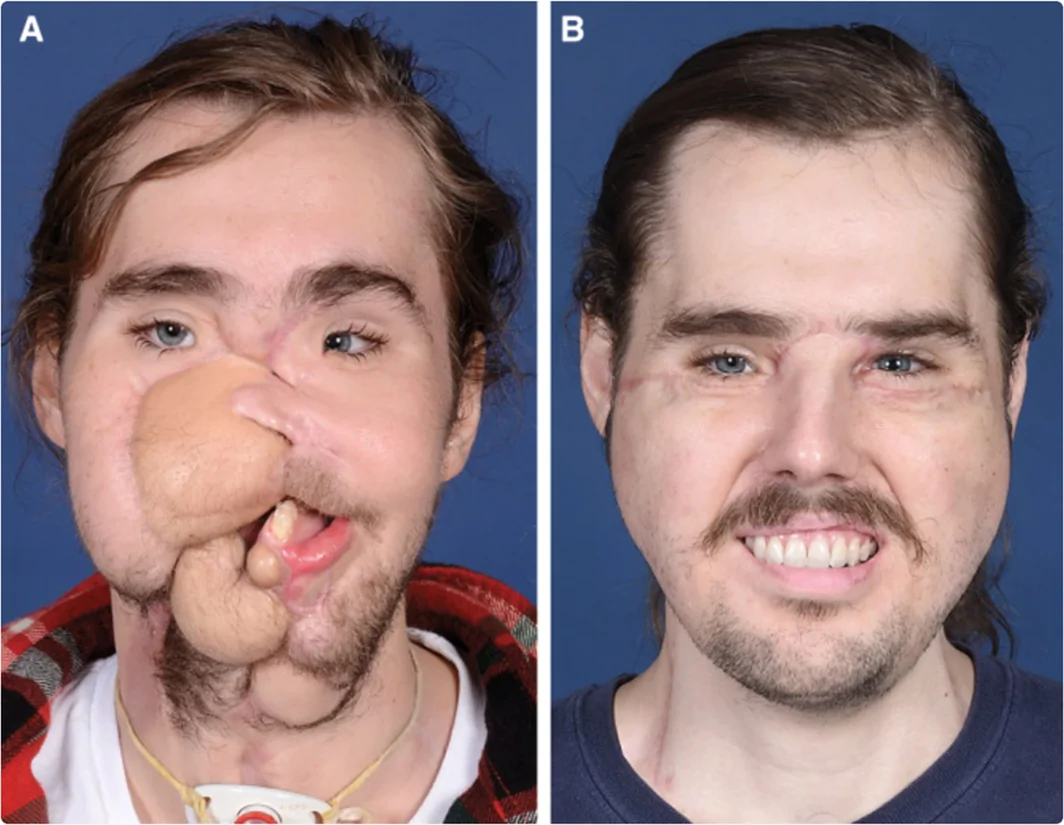
Recipient with disfiguring ballistic facial injuries before (A) and 9 months after (B) a partial face and double jaw transplant (Ramly et al. 2019). Reproduced from Ramly et al. (2019). 2019 The Authors. Copyrights retained by Eduardo D Rodriguez (E.D.R.). Permission granted by E.D.R. Licensed under the Creative Commons Attribution–NonCommercial–NoDerivatives 4.0 International License (CC BY‐NC‐ND 4.0). Available at: https://creativecommons.org/licenses/by‐nc‐nd/4.0/.

**FIGURE 2:**
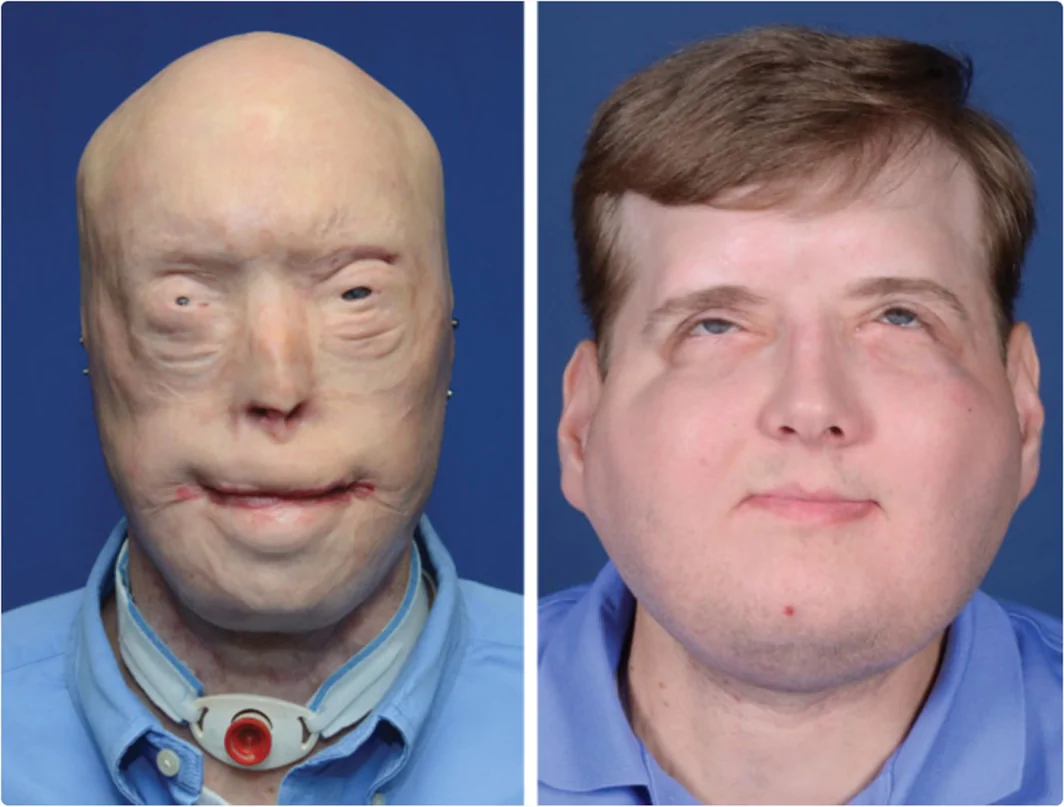
Recipient involved in a full‐face transplantation following severe burn injury (left) and after full‐face transplant (right) (Ramly et al. 2019). Reproduced from Ramly et al. (2019). 2019 The Authors. Copyrights retained by Eduardo D Rodriguez (E.D.R.). Permission granted by E.D.R. Licensed under the Creative Commons Attribution–NonCommercial–NoDerivatives 4.0 International License (CC BY‐NC‐ND 4.0). Available at: https://creativecommons.org/licenses/by‐nc‐nd/4.0/.

The procedure of facial transplantation begins with tissue being harvested bidirectionally from the donor in a craniocaudal and lateromedial direction (La Padula et al. 2022; Pomahac et al. 2012). The retrieval procedure is complex and can range between 10 and 30 h, requiring precise coordination of a vast multidisciplinary team, including surgeons, anesthetists, ophthalmologists, immunologists, surgical nurses, surgical technicians, and surgical photographers (Bueno et al. 2011; Eun 2015). The first partial FT was performed in France in 2005 (Giatsidis et al. 2017), and the first full FT in Spain in 2010 (Barret et al. 2011). To date, 50 FTs in 11 different countries have been recorded, with multiple indications, most commonly ballistic injury, burns, neurofibromatosis, and animal attacks (Homsy et al. 2024; Diep et al. 2020). The United States has performed the most FTs (16), followed by France (11), Turkey (8), and Spain (4) (Diep et al. 2021), with surgeons in the United Kingdom (UK) yet to attempt any form of FT. This is due to concerns about the associated psychological, ethical, and surgical complication risks being too substantial for a non‐life‐saving procedure (Bound Alberti and Hoyle 2022). Despite this, it is estimated that over 500,000 people in the UK alone live with a significant facial disfigurement which affects either their facial function, psychological well‐being, or both (Vehmas 2010). Although not appropriate for all, facial transplantation is an innovative surgical intervention with the potential to improve the quality of life for select individuals (Coffman et al. 2010).

The face contains key structures which need to be preserved and treated with care pre‐, peri‐, and post‐operatively, and so a sound anatomical knowledge is essential for an FT to be successful. In FTs, the key anatomical structures that need to be considered are the arterial supply, venous drainage, and the motor and sensory nerve supply to and from the face (Siemionow and Ozturk 2012). Understanding the locations, routes, and variations of these structures, a surgeon can maximize the precision of neurovascular re‐anastomoses and yield the best possible clinical outcome for the recipient (Prabhu et al. 2019).

Although it is also important to account for non‐neurovascular structures such as bone, soft tissue, and teeth (Diep et al. 2020; Palmer and Nelms 2020; Coombs et al. 2022), this review aims to discuss the neurovascular aspects of an FT, why they are important, and how they may be approached to maximize FT outcomes and success.

## 2 Materials and Methods

The database ‘PubMed’ was used for the initial search, using the following search term: “Face Transplant AND (allotransplantation OR anatomy OR donor OR graft OR neuro OR neurovascular OR outcomes OR reconstructive OR success OR surgery OR surgical approaches OR transplant OR vascularized composite allotransplantation OR vasculature).” A filter was set to contain articles from the year 2000 onward. The Boolean Operators, AND and OR, were used to keep results refined and specific. This gave a total of 9025 articles. The PubMed search result was saved and uploaded onto the software ‘Rayyan’, which detected 140 duplicates. These were subsequently removed. Initial screening of article titles left 606 accepted papers, only 83 of which were accepted after reading the abstract. After reading the articles in full, 18 final papers were accepted. These 18 papers provided a strong background on facial transplantation and inspired the structure of this comprehensive review (Figure 3). Despite this, due to the novel nature of this procedure, other articles found in manual searches, detailing the specifics of facial anatomy, other more routine procedures, and new experimental reports, were also relied on. This inspired and facilitated discussion on the possible ways to improve FT surgery. Wide access to full papers was enabled by the University of Leeds and its subscription to journals and OpenAthens membership.

**FIGURE 3:**
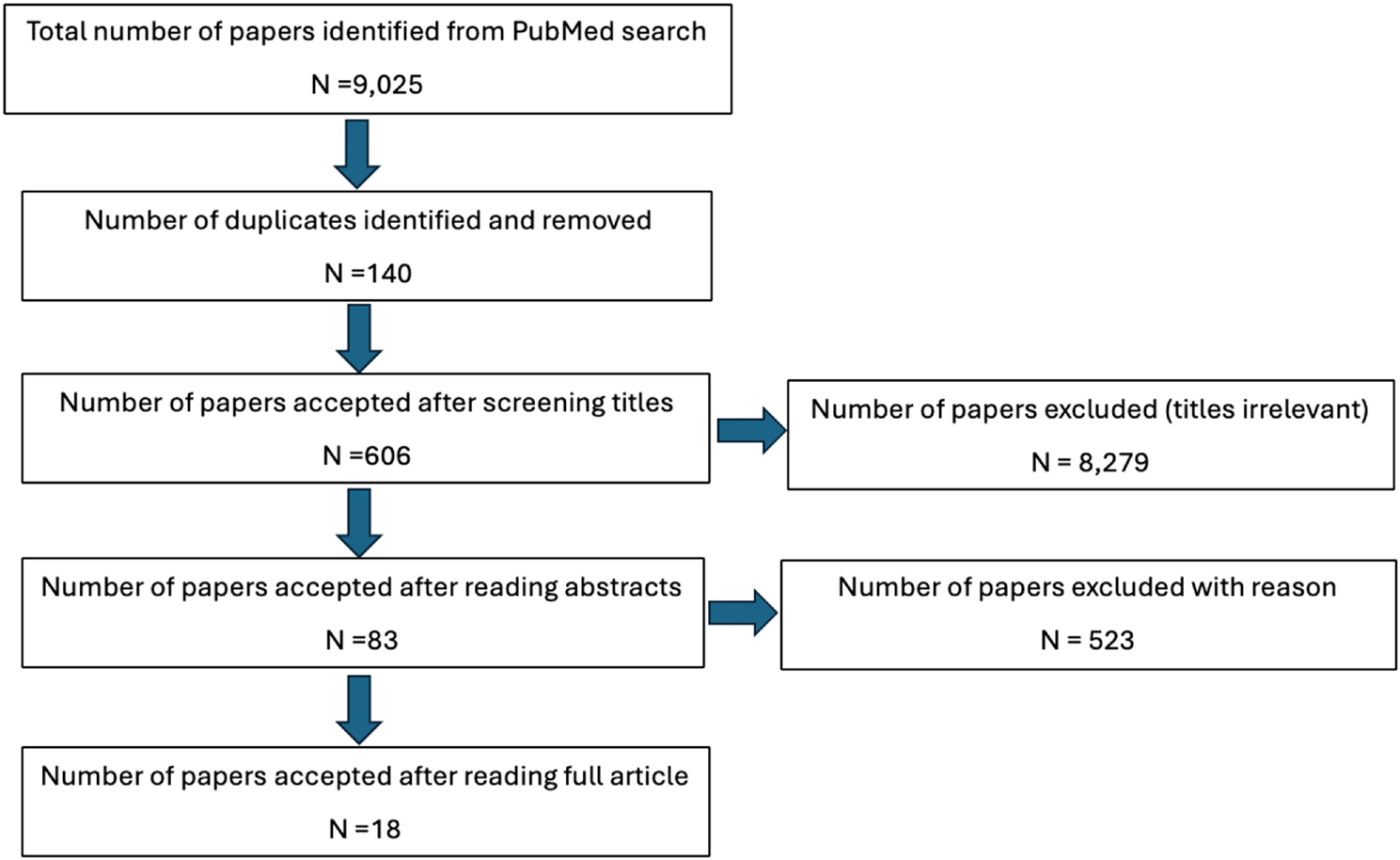
PRISMA chart showing how articles were identified and subsequently accepted or excluded.

Two detailed dissections of the head and neck were performed concurrently with the research and write‐up process. While no original cadaveric findings are discussed in this comprehensive review, original images of these dissections have been used to supplement the topics discussed where appropriate.

## 3 Results and Discussion

### 3.1 Vasculature of the Graft

#### 3.1.1 Arterial Supply and Venous Drainage of the Face

The blood supply to the head originates from the internal carotid artery (ICA) and external carotid artery (ECA), with branches from these arteries forming a highly anastomotic network throughout the face (Shihada et al. 2023). The majority of the face is supplied by the facial artery (FA) and the superficial temporal artery (STA), both of which take their origin from the ECA (Lohn et al. 2011) (Figure 4). The maxillary artery (MA), also a branch of the ECA, is the main supply to the deep structures of the face, including the maxilla and mandible (Gofur and Al Khalili 2023).

**FIGURE 4:**
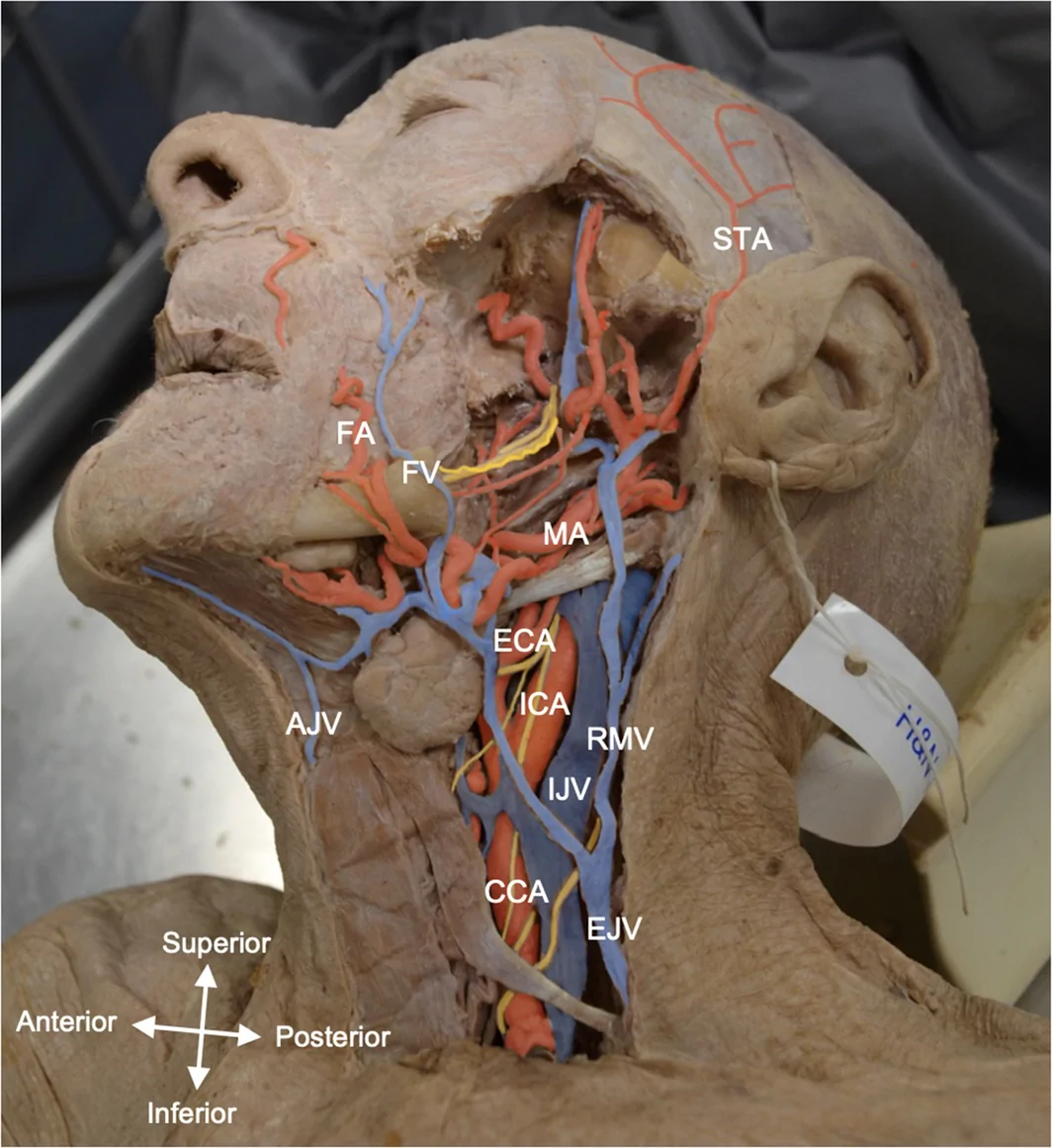
Left‐hand side deep dissection of the face and anterior triangle of the neck. The sternocleidomastoid muscle and the muscles of mastication have been removed. H&N 10/21. AJV, anterior jugular vein; CCA, common carotid artery; ECA, external carotid artery; EJV, external jugular vein; FA, facial artery; FV, facial vein; IJV, internal jugular vein; MA, maxillary artery; RMV, retromandibular vein; STA, superficial temporal artery; blue, veins; yellow, nerves; red, arteries.

The anterior jugular vein (AJV), external jugular vein (EJV), and internal jugular vein (IJV) are responsible for venous drainage of the head and neck into the subclavian veins (Isaac et al. 2025) (Figure 4). The AJV and EJV are found superficially and are responsible for the majority of the cutaneous venous drainage of the face. While the AJV receives mostly small, unnamed veins from the chin, the EJV receives contributions from multiple different tributaries, the facial vein (FV) and the retromandibular vein (RMV) being the most significant (Isaac et al. 2025) (Figure 4). The IJV drains mostly deeper structures of the head, including the brain and skull (Rivard et al. 2025).

#### 3.1.2 Relevance of Arterial Supply and Venous Drainage in Facial Transplantation

Constructing an effective vascular supply is critical to prevent graft necrosis and graft loss (Alam 2014). As there is high person‐to‐person variability in head and neck vascular anatomy, it is important to build a clear picture of both donor and recipient vasculature before an FT operation (Tan et al. 2010). This helps ensure the desired vessels are surgically accessible and that the donor and recipient vessel lumina are appropriately matched in size. It is also key to avoid excessive manipulation of vessels. When vessels are anastomosed under high tension, the pull on the sutures can result in damage to the vascular wall, bleeding, vasospasm, and ultimately thrombosis or dehiscence (Yang and Chung 2018). Awareness of vasculature anatomy allows the vessels in close proximity to be chosen, preventing the stretching or pulling on vessels, helping aid a successful, low‐tension anastomosis (Soga et al. 2013). Additionally, knowledge of vessel location and route can prevent accidental peri‐operative damage (Prabhu et al. 2019).

Many FT candidates have suffered from severe facial trauma and may have already undergone reconstructive attempts, which increases the chances of the patient presenting with a complex, atypical vascular anatomy following neovascularization (Soga et al. 2013). With this knowledge prior to operating, vessels with damage or unfamiliar characteristics can be excluded from the graft, as they may be unable to sufficiently support the transplanted tissue. If the first line choice of vessel, usually the FA, is not suitable or cannot be accessed, anatomically inferior arteries away from the trauma site, such as the ECA or the CCA, can be used (Diep et al. 2021; Von Arx et al. 2018).

Pomahac et al. (2012) concluded that the FAs alone are sufficient to support even a full FT. This conclusion was based on three successful FTs using only bilateral facial vessels to support the graft. Although there is some reproducibility with this method, the suggestion is somewhat limited as it is not based on statistical, clinical, or experimental analysis. And while this conclusion may be accurate, a functioning vascular anastomosis is critical for FT success; therefore, at least one additional vessel, most commonly the STA, is usually also connected to act as a safety net (Soga et al. 2013; Nguyen et al. 2013). Nevertheless, the study by Pomahac et al. (2012) does match the recommendations of multiple other research papers, that the FA should be prioritized as the main arterial pedicle where appropriate, due to its predictable route and high blood flow (Lohn et al. 2011; Garg et al. 2017; Renshaw et al. 2007). Renshaw et al. (2007), made this recommendation based on the study of 200 FAs in 100 individuals, which located the FA at the lower mandibular border in 99.5% of cases. This high reproducibility adds value to the recommendation.

As FTs mostly consist of the superficial facial material, the MA is rarely the primary vessel of anastomosis. Furthermore, its deep and complex route makes it a difficult vessel to harvest (Gharb et al. 2014). Touré and Meningaud (2015) injected India ink into the FAs of 22 excised maxillae and concluded that the FA could provide a sufficient arterial supply to a graft containing the maxilla through its collateral branches, specifically the superior labial artery and the ascending palatine artery. This observation is valuable and may be applied, with caution, to more extensive FTs which require maxilla transplantation, reducing the need to access the MA and subsequently preventing the disturbance and damage of deeper facial structures.

A study conducted by Nahabedian et al. (2004) analyzed the different causes of flap necrosis in microvascular breast reconstruction following mastectomy. The outcomes of 240 flaps were followed, demonstrating that venous occlusion was the most common cause of graft failure and was significantly associated with the need to return to the operating room (*p* < 0.001). Soga et al. (2013) paired this early research with other data focusing on head and neck microsurgical reconstruction to conclude that at least two venous anastomoses should be used to ensure sufficient drainage of an FT graft (Kubo et al. 2002; Pohlenz et al. 2012). Papers over the last 35 years consistently recognize inadequate venous drainage as a leading cause for microvascular flap failure (Suh et al. 2021). Consequently, this recommendation of Soga et al. (2013) is based on reproducible and reliable data and thus should be strongly considered.

Despite the importance of adequate venous drainage, it is interesting to note the lack of research into which specific venous pedicles should be used, especially in comparison to the abundance of studies focusing on the recommended arterial pedicles. Most studies comment that a successful venous outflow can be achieved using FVs, RMVs, and EJVs (Bondaz et al. 2014; Nguyen et al. 2013). Rodriguez‐Lorenzo et al. (2015) commented that due to the worry of venous occlusion, surgeons will usually perform multiple venous anastomoses for a graft, meaning it is difficult to establish the significance of each vein in supporting the graft. To evaluate if a single FV was sufficient to support a total FT, Rodriguez‐Lorenzo et al. (2015) worked with five human cadaveric full‐face flaps. A single FV on each flap was injected with radiopaque contrast, and three‐dimensional computed tomographic venographies were taken (Figure 5). These images were used to conclude that while there is a high anastomotic network between the left and right FVs, there is a significant reduction in perfusion of the contralateral cheek and lower face. Therefore, it could be suggested that bilateral venous anastomosis should be performed where possible to ensure adequate venous outflow of a full FT. Given the lack of alternative literature on this topic, coupled with the thoroughness of this study, this is one of the most insightful and valuable papers regarding venous grafts within the face to date. Although the research does not discuss partial FTs, it may be hypothesized that while one FV is insufficient for a full FT, it may be adequate to support and drain a partial FT, where the graft only involves one side of the face.

**FIGURE 5:**
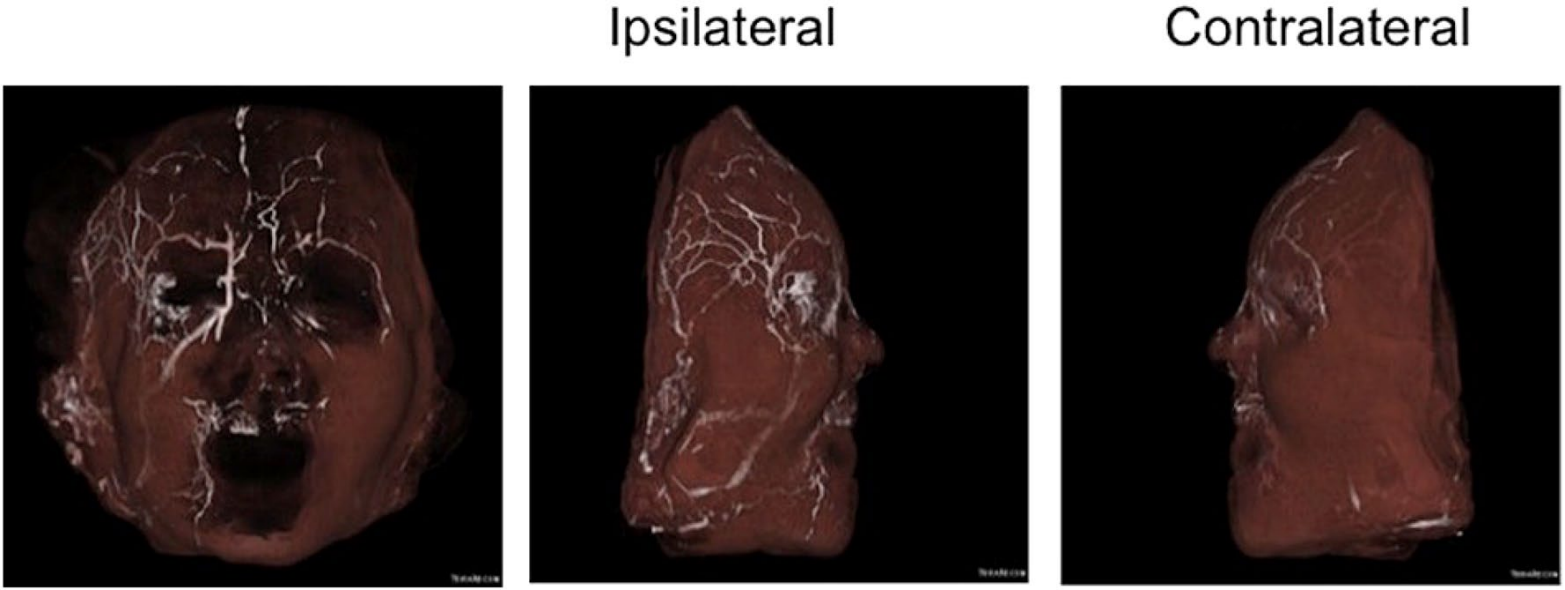
Venography of 1 of the 5 face‐flaps injected with radiopaque contrast, showing decreased venous perfusion in the contralateral cheek, lower lip, and chin (Rodriguez‐Lorenzo et al. 2015).

#### 3.1.3 The Importance of Pre‐Operative Identification of Vessels

Three main methods of pre‐operative vessel identification have been proposed. These include computed tomography angiography (CTA), magnetic resonance angiography (MRA), and digital subtraction angiography (DSA) (Prabhu et al. 2019; Soga et al. 2011; Renshaw et al. 2007). Each method has its strengths and limitations.

Soga et al. (2011) compared four‐dimensional CTA and MRA. Both imaging techniques involve the injection of intravenous contrast, opacifying vessels on delayed imaging sequences (Mazonakis and Damilakis 2016; Kuo et al. 2019). The study found that CTA was significantly better at visualizing small vessels (*p* < 10^−4^), and far less affected by metal artifact, most commonly caused by previously implanted surgical clips or prostheses (Figure 6) (Soga et al. 2011). These findings led Soga et al. (2011) to suggest that CTA should be used first line in vascular mapping prior to an FT. Soga et al. (2011) drew this conclusion after imaging 126 vessels (76 arteries, 50 veins) across the faces of five individuals with both imaging modalities, giving it a high statistical power. Other, non‐FT specific papers which analyzed coronary and intracerebral CTA versus MRA also concluded CTA superiority (Sakuma 2011; van Asch et al. 2015).

**FIGURE 6:**
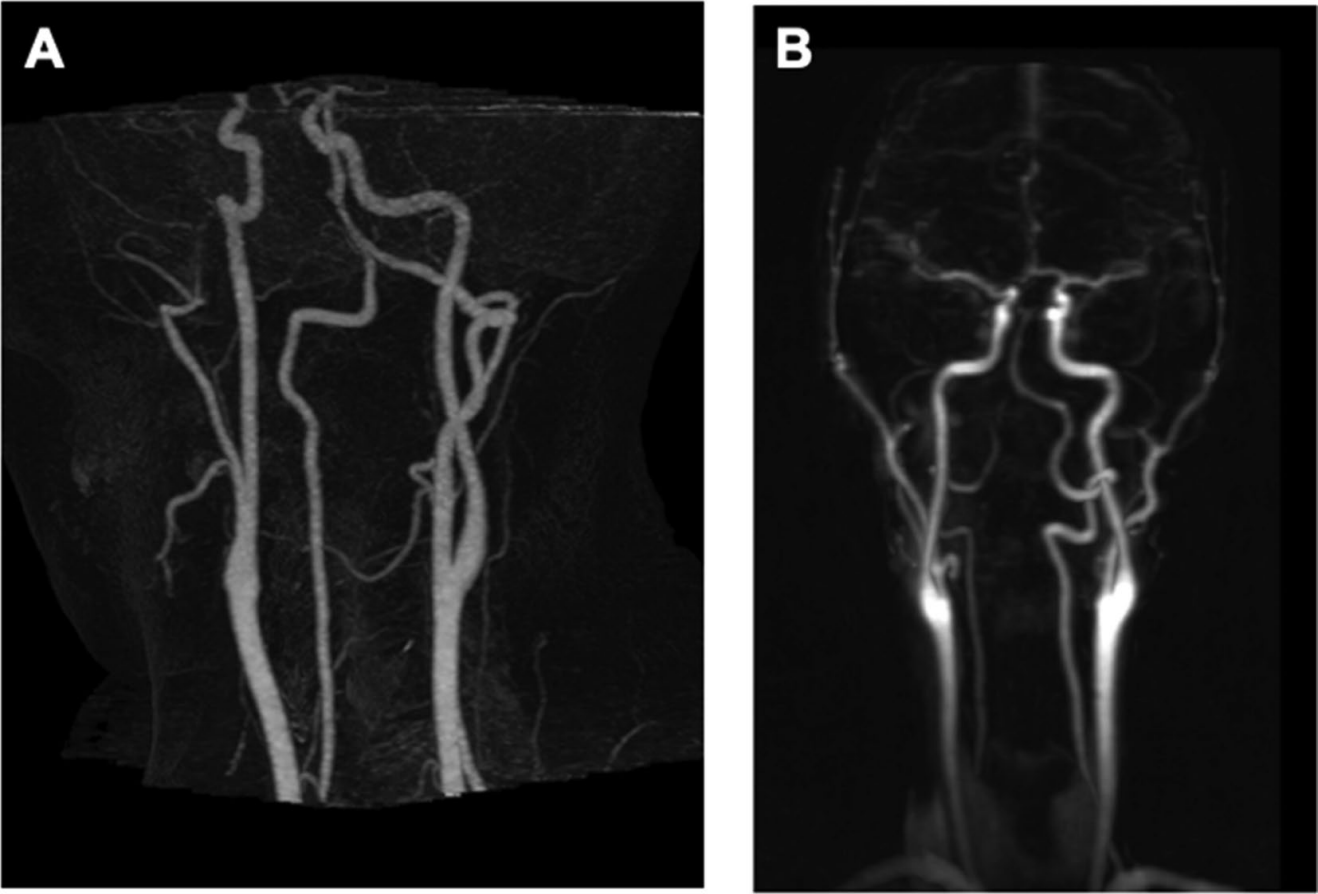
Detailed mapping images from an arterial computed tomographic angiography (CTA) (A) and magnetic resonance angiography (MRA) (B); adapted from (Soga et al. 2011).

Although CTA appears to be the superior imaging modality of vasculature, it does come with several radiation exposure risks due to its ionizing properties. This includes the development of brain cancer, with a patient being 50 times more likely to develop radiation‐induced brain cancer after undergoing a head and neck CTA (Ivanidze et al. 2017). Consequently, considerations should be made when deciding on the most appropriate imaging modality to assess the facial vasculature of a patient. For example, conducting a risk assessment on FT recipients, which could identify an increased susceptibility to radiation‐induced malignancies, for example, increasing age and a positive individual or family history of cancer (Morton et al. 2014). In high‐risk cases, it may be better to opt for non‐ionizing MRA imaging, a safer alternative for the patient.

Much like CTA and MRA, DSA requires the use of a peripheral injection of intravenous contrast (Johnson et al. 1984). Two images are captured by X‐ray: one with the contrast and one without. The two images are then digitally subtracted from each other, removing unwanted background structures like bone and soft tissue, leaving a clear image of the blood vessels filled with contrast (Figure 7). The final image provides valuable information on the route and flow of blood through vessels, allowing identification of blockages or anatomical abnormalities in the vasculature (Scholz 1986).

**FIGURE 7:**
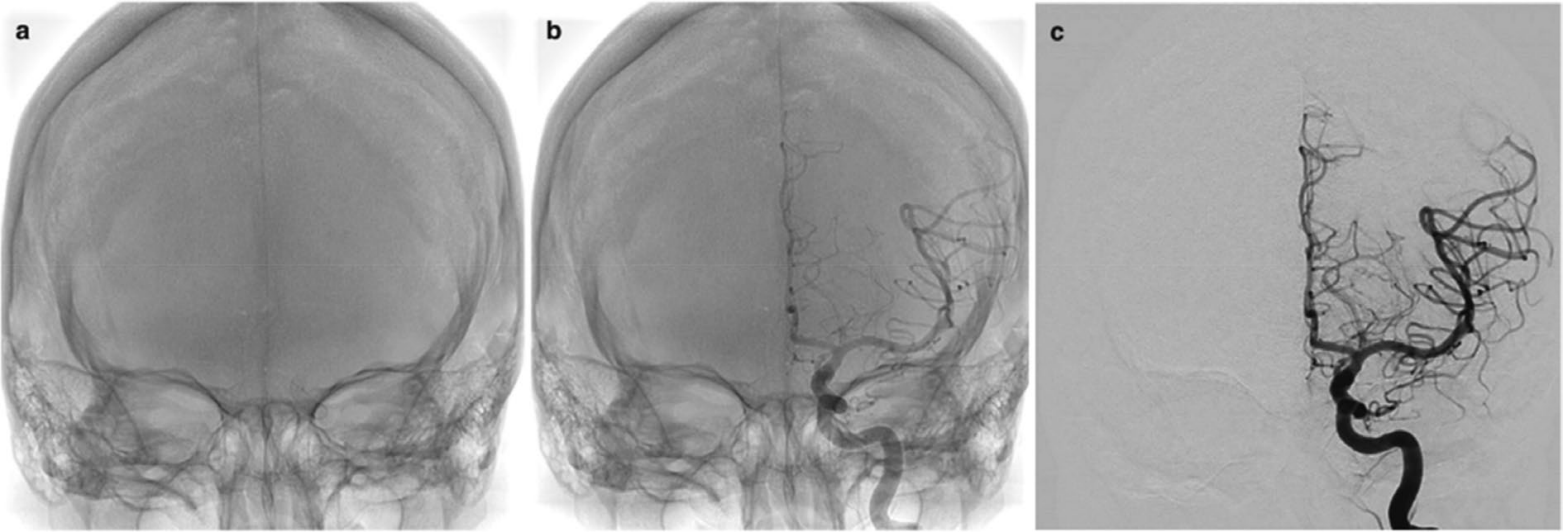
Digital subtraction angiography (DSA) imaging of the internal carotid artery; adapted from (Hendriks et al. 2020). Head X‐Ray before contrast (a), after contrast (b), and after a and b are digitally subtracted (c).

The role of DSA in FTs was discussed by Prabhu et al. (2019), who used DSA to assess the vasculature of the ECAs and their branches in an individual who had suffered a high‐energy ballistic facial injury. The images showed evidence that the patient's tongue was unilaterally supplied by the right lingual artery (RLA), information that had not been detected on the patient's earlier CTA. With a more accurate knowledge of this unique vasculature provided by DSA, an extremely careful, uneventful dissection around the RLA was performed. Damage to this vessel could have led to post‐operative tongue necrosis. Following their investigation, Prabhu et al. (2019) suggested that DSA imaging should be a fundamental component of pre‐operative FT imaging as it can ensure more informed intra‐operative vasculature management, resulting in improved patient outcomes.

These conclusions were corroborated by a recent study, which referred to DSA as the ‘gold standard’ imaging modality in pre‐operative arterial analysis (Manasyan et al. 2025). This report analyzed the angiographies of 175 patients awaiting lower extremity reconstruction with an autogenous free flap. While for the majority of cases, CTA was adequate, an additional DSA was required in 11 patients due to insufficient or nonspecific findings. In five of these, the DSA showed new information detailing missed arterial disease and damage. Manasyan et al. (2025) concluded that although the more cost and time‐effective CTA is mostly suitable, if results are inconclusive or limited, DSA should then be employed for further evaluation. This literature on DSA superiority is both modern and reproducible. It would be reasonable to infer that while it need not be first line, DSA should be an accessible imaging modality in the arterial work‐up of FT patients.

Despite its ability to detect complex vasculature, due to the nature of DSA, it does not provide information on the relationship of vessels with the surrounding soft tissue and bone (Tobis et al. 1983). Knowledge of these structures and their relationship with the arteries can be key for uneventful vessel identification and dissection (Prabhu et al. 2019). This is particularly relevant in FT candidates, as many have vessels embedded in scar tissue due to post‐traumatic and post‐surgical adhesions (Fatehi Hassanabad et al. 2021). Therefore, DSA should be used to complement CTA and MRA, rather than in place of them (Prabhu et al. 2019; Kassem et al. 2021).

### 3.2 Optimizing Vessel Anastomosis

End‐to‐end and end‐to‐side vessel anastomosis are two types of microsurgery commonly involved in microvascular free tissue transfer (Vila et al. 2020). End‐to‐end involves lining up the naturally occurring vessel lumina from the recipient and the graft, whereas end‐to‐side involves creating a new opening in the side of a vessel wall (Ahmed and Gilhooly 2017). Each method has advantages and limitations depending on the type and the structure of vessels being joined (Samaha et al. 1997). In FTs, end‐to‐end anastomosis is considered to be a simpler approach and is often used first line for joining straight vessels such as veins, for example, the FV, which has a relatively linear course (Siwetz et al. 2023). Using end‐to‐side anastomosis for these vessels would create new, unnecessary changes to their direction, which can increase intravascular blood turbulence and predispose the vessel to damage, platelet aggregation, and subsequent thrombosis (López‐Monjardin and PEÑA‐Salcedo 2000).

Alternatively, despite being considered a more complex procedure, the end‐to‐side technique is often more appropriate for anastomosing arteries, as many of the ECA branches are tortuous due to their higher intravascular blood pressure (Han 2012). This can make it difficult to directly line up vessel lumina. Furthermore, this technique is advantageous when there is a discrepancy between vessel lumen size, which, if connected, is at an increased likelihood of altered flow, anastomotic leaks, and thrombotic events (Izadpanah et al. 2024). End‐to‐side is also appropriate if the end of a vessel is damaged (Vila et al. 2020). This is a common challenge in FT candidates, as many have suffered from facial trauma, which can impair vessels that might later be needed to support a graft (Soga et al. 2013; Vila et al. 2020).

Cavadas et al. (2012) reported the case of a partial, lower FT. It was the first of its kind to successfully incorporate the tongue, floor of the mouth, and most of the mandible. They noted the use of a simple and rapid end‐to‐end anastomosis to join the recipient's and donor's right IJVs and ICAs. A more timely and complex end‐to‐side anastomosis was used to join the recipient's right subclavian artery to the donor's right CCA. This study reinforced that for a successful FT, different methods of vessel anastomosis should be practised according to vessel route and requirements. Despite only focusing on one FT, this report provides valuable insight into the potential best practice of vascular anastomoses in FT. As only 50 human FTs have currently been performed, it is difficult to heavily critique the use of a small sample size. At the time of publication, it was difficult for Cavadas et al. (2012) to comment on the reproducibility of these findings; however, with new research discussed above, it is reasonable to consider this a reliable conclusion (Vila et al. 2020; López‐Monjardin and PEÑA‐Salcedo 2000).

In both anastomotic methods mentioned, vessels are typically joined using an open‐loop suturing technique (Vila et al. 2020). This time‐efficient method is a form of continuous suture, which, through the creation of loops, allows visualization of the vessel lumen before the final stitch is made, ensuring patency of the new vessel (Figure 8) (Hodea et al. 2025). Abdelsayed et al. (2022) analyzed the advantages of open‐loop suturing in vessel anastomosis compared to interrupted suturing (Figure 9), which was traditionally used to join vessels. To explore this, the femoral arteries of 20 rats were incised bilaterally and then re‐anastomosed using the two techniques (one on the left and the other on the right). Using an ultrasonic Doppler to assess blood flow at 5 and 15 min, the study concluded there was no significant difference in vessel patency or anastomotic leakage between suturing techniques. The anastomotic time of each method was also recorded, and the means calculated; the conventional interrupted sutures took an average of 24.50 min, compared to the open‐loop technique, which took 22.40 min. Based on these findings, Abdelsayed et al. (2022) suggested that although continuous and interrupted suturing are both suitable, safe choices for anastomosing vessels, open‐loop sutures should be favored, especially when multiple different anastomoses are required. As FTs are already heavily time‐consuming procedures, this seems like a suitable suggestion.

**FIGURE 8:**
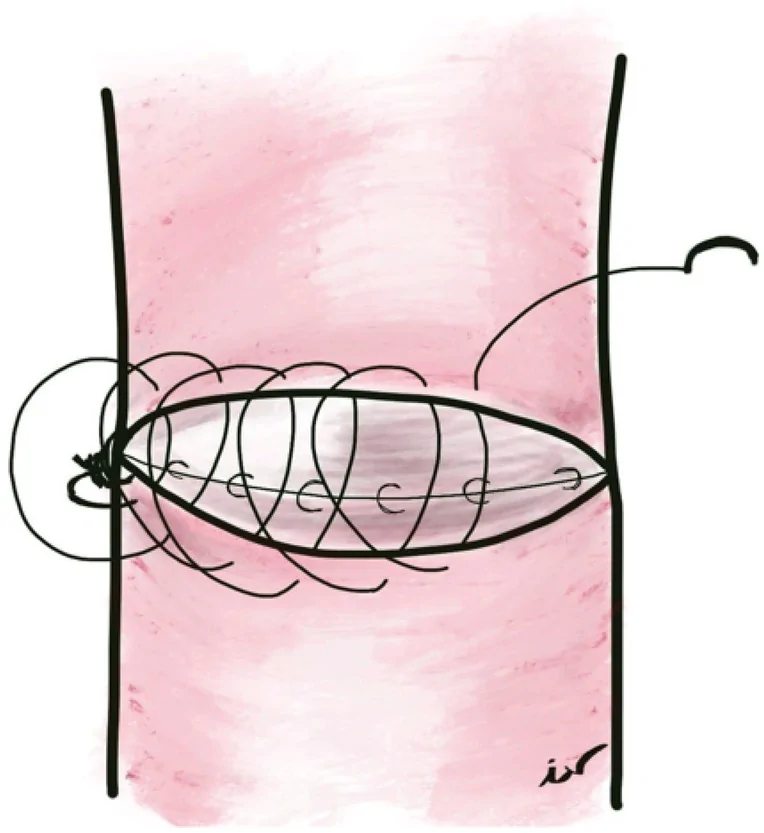
Open‐loop suture technique anastomosing two vessels in an end‐to‐end fashion (Vila et al. 2020).

**FIGURE 9:**
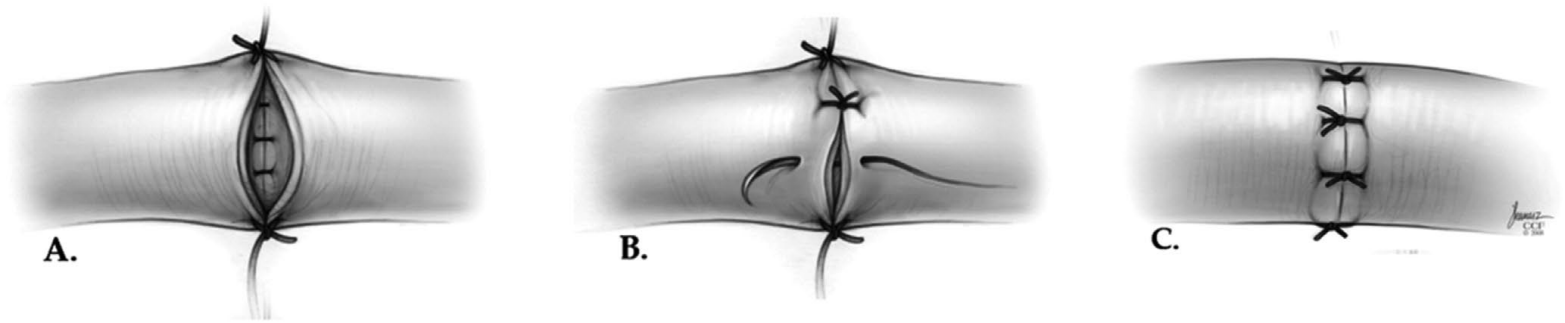
Conventional interrupted suturing technique anastomosing two vessels in an end‐to‐end fashion. Adapted from Alghoul et al. (2011).

It is important to note that the results of Abdelsayed et al. (2022) were not from human surgical cases. The mean diameters of a rat femoral artery and a human FA are 0.54 mm (Liu 2013) and 1.20 mm (Lee et al. 2019), respectively, making the human FA over twice the diameter of a rat's femoral artery, on average. This is important to consider when applying the findings of these studies to human vasculature suturing, as lumen size can affect the integrity of a suture due to the varying pressure and resistance exerted by the vessel (Mulvany 1999). This reduces the relevance of the studies regarding their ability to be directly applied to FTs. However, this type of research would be unlikely to gain ethical approval in human patients.

### 3.3 Nerves of the Graft

#### 3.3.1 Motor and Sensory Innervation to the Face

Sensory innervation to the face is supplied solely by cranial nerve V, the trigeminal nerve. This cutaneous innervation is distributed in three key branches: the ophthalmic nerve (V1), the maxillary nerve (V2), and the mandibular nerve (V3) (Walker 1990). Cranial nerve VII, the facial nerve (FN), provides motor innervation to the muscles of facial expression (Figure 10) (Sanders 2010). These are the most superficial muscles of the face and are almost always included in an FT graft (Fischer et al. 2015). The motor root of the FN exits the cranium via the stylomastoid foramen and continues anteriorly through the parotid gland, where it gives rise to five main divisions: temporal, zygomatic, buccal, marginal mandibular, and cervical. The muscles of mastication are located deeper in the face and are innervated by motor branches of the mandibular nerve (V3) (Sanders 2010). Incorporation of these muscles into an FT graft is common, but not a universal requirement, and thus will depend on the location and depth of the transplant (Pomahac et al. 2014).

**FIGURE 10:**
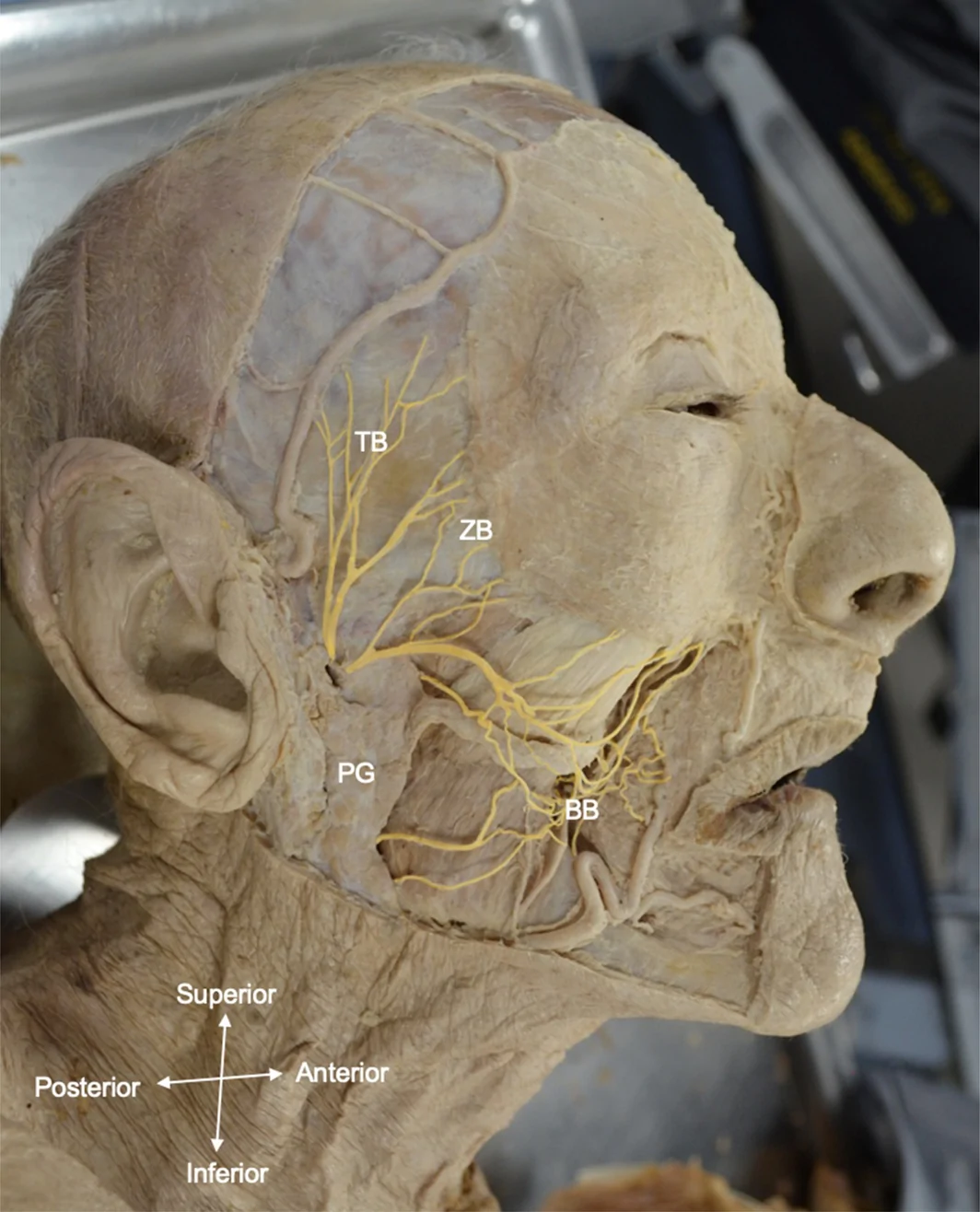
Right‐hand side superficial dissection of the face to show the intricate branching pattern of cranial nerve VII, the facial nerve. Branches of three of the five main divisions are visible: temporal, zygomatic, and buccal. H&N 10/21. Yellow, branches of the facial nerve (FN). BB, buccal branches; PG, parotid gland; TB, temporal branches; ZD, zygomatic branches.

#### 3.3.2 Relevance of Motor and Sensory Innervation in Facial Transplantation

Sensory innervation has a significant role in the way an individual experiences an environment. Touch, temperature, and pain sensation from the face provide the brain with information which is key in basic survival, as well as facilitating social interactions and improved well‐being (Liu et al. 2019). Thus, establishment of good sensory innervation to the transplanted graft is essential. Surgical reconstruction of the trigeminal nerve's cutaneous branches is variable throughout the literature. In some FT cases, the donor and recipient branches were repaired and joined (Devauchelle et al. 2006). In others, the nerves were left unattached. This was mainly due to trauma‐related shortening and damage of the recipient's nerve, meaning coaptation was not feasible (Guo et al. 2008; Siemionow et al. 2009). Interestingly, there was no significant difference in outcomes between the two methods, and nearly normal sensory recovery could be achieved with or without surgical nerve repair (Siemionow et al. 2011b). Spontaneous nerve recovery, without surgical intervention, is thought to occur due to the dense sensory nerve plexus found in the facial area, which allows for accelerated regeneration across the donor–recipient tissue boundary (Siemionow et al. 2011a). Since 2012, there has been a lack of new research to test or challenge these conclusions in the literature. Most of the research discussed focused specifically on the immediate and short‐term success of sensory nerve recovery (Siemionow et al. 2011b). It is possible that more long‐term follow‐up data collection is underway and has yet to be reported and published.

Contrary to the findings regarding sensory reinnervation, restoration of good motor nerve function is challenging and requires much more active surgical manipulation and management (Aycart et al. 2016). Adequate motor function is essential to achieve the fundamental FT aims: improved functionality and appearance of the face (Bound Alberti and Hoyle 2022). However, to restore any level of function, branches of the graft's motor nerves need to be coaptated with the recipient's (Shokri et al. 2020). The approach to achieving optimal FN recovery has been of particular interest and importance, especially as the loss of facial animation is strongly associated with depression and social isolation (Boahene 2013; Frautschi et al. 2016). While recipients are already predisposed to psychological challenges following even a successful FT (Huelsboemer et al. 2024), failure to restore FN function would severely limit the functionality of the graft and subsequently the recipient's quality of life.

#### 3.3.3 Preservation and Effective Coaptation of the Facial Nerve

Inclusion versus exclusion of the parotid gland in the FT graft has been debated when considering maximizing FN recovery (Frautschi et al. 2016; Diep et al. 2020; Alam 2014; Pomahac et al. 2012). In either case, the recipient gland is usually left in situ regardless (Frautschi et al. 2016). Inclusion of the donor parotid gland allows for a simpler coaptation of the FN at its trunk, whereas exclusion of the parotid gland is more complex, requiring careful dissection around the graft's gland, followed by coaptation of the individual branches (Pomahac et al. 2012). Frautschi et al. (2016) reviewed the cases of 39 FN repairs using allografts; 23 involved the whole parotid gland, two involved only the superficial lobe of the gland, and 14 excluded the entire gland. Although there was no statistically significant superiority of any method in regard to post‐transplantation FN recovery and function, the study noted repetition in the concern regarding complications of parotid gland inclusion, including excessive saliva accumulation, salivary stone formation (sialoliths), and undesirable facial bulking (Norrman et al. 2021; Lee et al. 2015). Saliva contains autolytic enzymes which, when stagnant, increase the risk of fistula formation and infection, which subsequently compromise wound healing (Harrison 2009; Parekh et al. 1989). Therefore, it might be beneficial to exclude the donor parotid gland from the graft, even if not directly related to FN recovery and function.

Methods of direct FN coaptation can vary. Most commonly, FN ends are anastomosed using the finest gauge of microsurgical nylon suture, typically 10‐0 sutures, fibrin glue, or both in combination (Figure 11) (Bento et al. 2008; Sánchez‐Ocando et al. 2019; Alejandro and Jesus 2022; Jandali and Revenaugh 2019).

**FIGURE 11:**
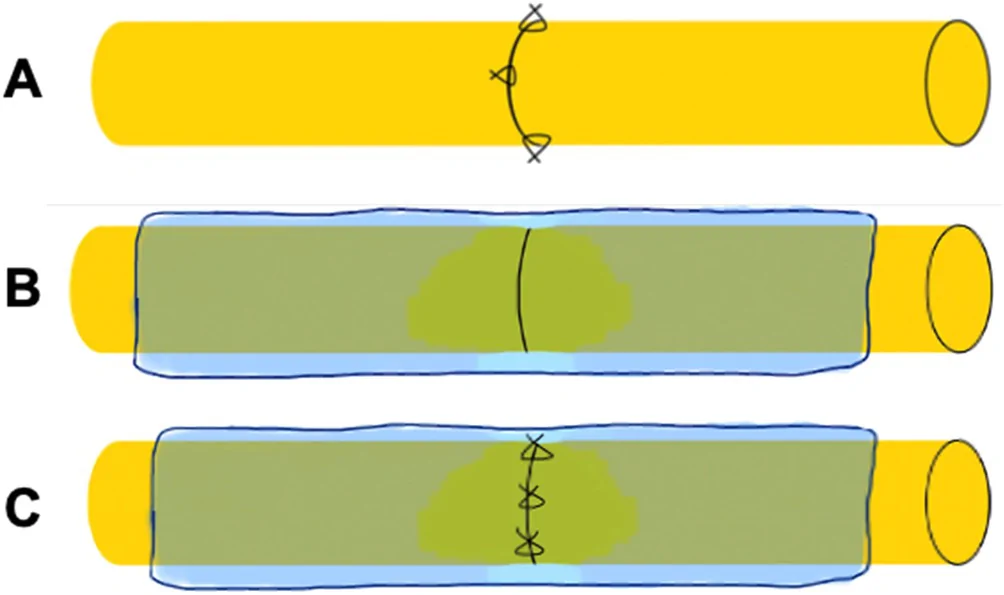
Coapting nerves in an end‐to‐end fashion using only sutures (A), only fibrin glue (B), and sutures and fibrin glue (C); adapted from Wang et al. (2018).

The use of fibrin glue alone initially seems advantageous as technically easier, quicker, and reduces the amount of direct trauma to the nerve (Koopman et al. 2022; Knox et al. 2013), coupled with the suggestion that complications of sutures include inflammation and scar formation at the suture site, which hinder nerve regeneration (Atkins et al. 2006). This assumption was analyzed by Sánchez‐Ocando et al. (2019), who looked retrospectively over 17 years at the cases of 36 patients who had undergone FN repair for various underlying diseases. Micro‐sutures in combination with fibrin glue were used in 17 cases; the other 19 cases used fibrin glue alone. The success of FN recovery was determined using the House‐Brackmann (HB) scale, scored I‐IV, and the electronic clinician‐graded facial function grading system, scored 0–100. Both tools are found to be accurate and reliable methods of assessing FN function (Reitzen et al. 2009; Banks et al. 2015). Despite the cases with sutures achieving higher, more consistent HB grades, no significant difference, defined as *p* < 0.05, was found between the success of the two methods (*p* = 0.069). Nevertheless, it was recommended that sutures and glue should be used together where possible (Sánchez‐Ocando et al. 2019). The reasoning for this is that, if nerve shift or malalignment were to occur, facial paralysis is a possible complication (Huang et al. 2023).

This caution is validated by a meta‐analysis performed by Koopman et al. (2022). The study analyzed 43 articles, each reporting the outcomes of various nerve repairs using fibrin glue or a combination of fibrin glue and sutures. The articles included data from 37 animal, two cadaveric, and four human studies. It was identified that nerves joined using glue alone had decreased stability and higher avulsion rates, especially following early post‐operative mobilization (Reza et al. 2014; Chao et al. 2001; Sames et al. 1997). Consequently, it would seem appropriate to conclude, if time and resources allow, that the two methods should be used together to maximize nerve stability and reduce the likelihood of dehiscence (Koopman et al. 2022).

#### 3.3.4 Methods of Improving and Enhancing Nerve Recovery

On occasion, a direct, tension‐free coaptation of graft and recipient motor nerves is not possible. This is usually the result of initial facial disfigurement or previous reconstruction attempts, which have subsequently led to scar formation, making the length and/or location of donor and recipient nerves, most commonly the FN, incompatible (Audolfsson et al. 2013). In these cases, nerve grafts harvested from the recipient can be used to bridge the gap between the graft and the recipient nerve branches (McQuillan et al. 2024). This is known as neurotization and can increase the likelihood of successful nerve recovery (Meals and Nelissen 1995).

Multiple articles suggest that the thoracodorsal nerve (TDN) is a suitable graft to use for FN neurotization (Aycart et al. 2016). This recommendation has been made on case‐based surgical evidence, where the TDN was used to successfully bridge large FN gaps (Biglioli et al. 2014; Bedarida et al. 2020; White et al. 2006). This is likely to be because the TDN is a close anatomical and functional match to the FN (Figure 12). They are similar in branching pattern and size, and both have a mean diameter of 2.5 mm (Zin et al. 2012; Khoa et al. 2019). Additionally, both provide solely motor innervation (White et al. 2006). The great auricular nerve (GAN) has also proven successful in several FN neurotization procedures outside of the FT setting following traumatic, inflammatory, and iatrogenic injuries to the FN (Sun et al. 2015; Werner et al. 2021; Altafulla et al. 2019). Thus, in cases of TDN unavailability, the GAN could be considered as an alternative graft source. The use of other nerves, including several cranial nerves, the sural nerve, and branches of the cervical plexus has also been proposed (Siemionow et al. 2010; Dorafshar et al. 2013; Altafulla et al. 2019; Sun et al. 2015). However, these recommendations are generally based on cadaveric studies or isolated successful cases, which may lack reproducibility and applicability to in vivo FTs. It may be beneficial to the future of FT surgeries if a more extensive, comparative study between the TDN and the GAN were performed to help determine the best graft source for FN neurotization.

**FIGURE 12:**
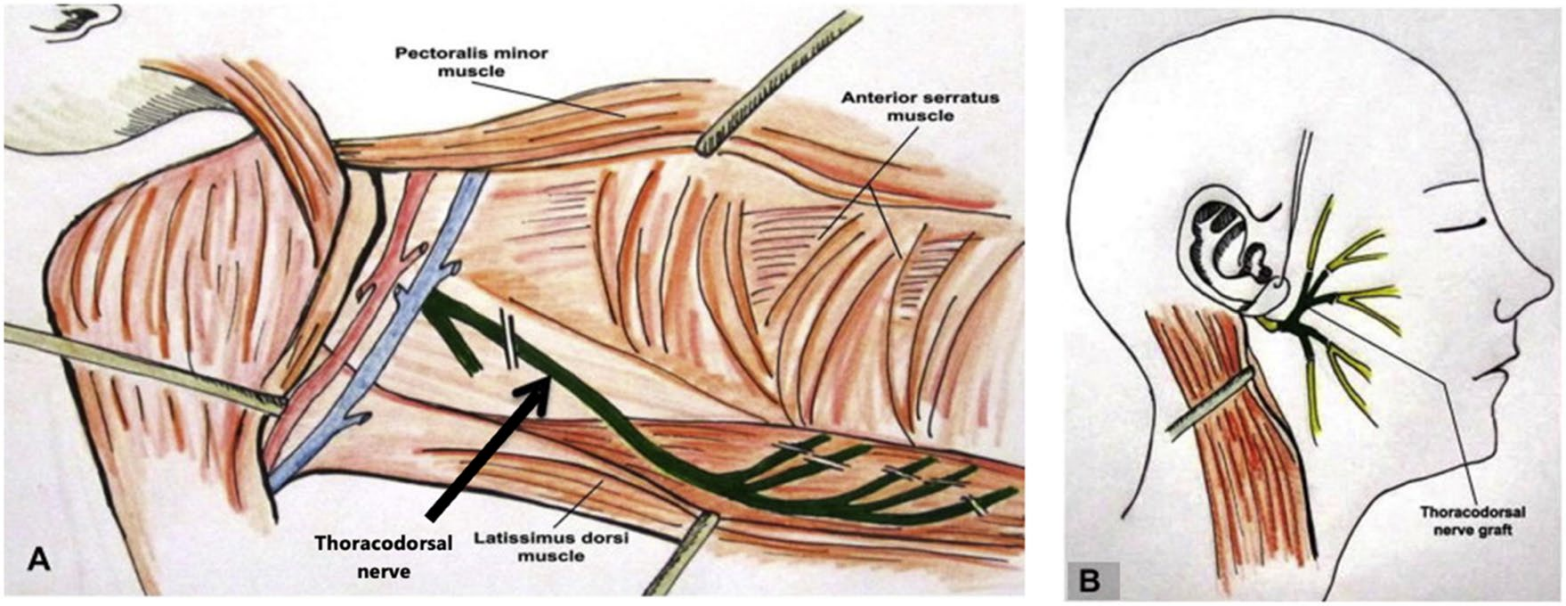
Thoracodorsal nerve (TDN) graft procedure. The thoracodorsal nerve is isolated and harvested along with its main branches (A), then transplanted to bridge the gap between the facial nerve trunk and its branches (B); adapted from Biglioli et al. (2014).

The rate of nerve regeneration is another critical contributor to FT success. Slow nerve recovery delays reinnervation of graft components, which can result in irreversible atrophy of graft muscle and tissue (Tung 2015). One proposed method of enhancing the speed and extent of motor and sensory nerve recovery is the application of topical tacrolimus (a calcineurin inhibitor) to the graft post‐operatively (Tung 2015, 2010; Pomahac et al. 2013). Although tacrolimus was initially prescribed to FT recipients as an oral immunosuppressant to prevent allograft rejection, the neuroprotective and regenerative effects were a secondary serendipitous finding (Pomahac et al. 2013). There is profound evidence to support the neuroregenerative properties of tacrolimus. A meta‐analysis of 22 studies found a statistically significant effect of tacrolimus on peripheral nerve regeneration (*p* < 0.01) (Seixas et al. 2022). Meta‐analyses possess high statistical power and precision due to their large sample sizes; thus, the findings of this paper can be considered highly reliable.

However, detrimental side effects of this drug, including nephrotoxicity, hyperglycemia, and hypertension, mean that systemic administration for immunosuppression is now rarely used (Konofaos and Terzis 2013; Tung 2010; Yang et al. 2003). Tacrolimus toxicity is dose‐dependent; therefore, these complications may be avoided when used topically (Yang et al. 2003). Despite this, due to its narrow therapeutic window, clinicians are still reluctant to prescribe its topical form without further research (Millesi et al. 2024). Two recent studies have explored the possibility of administering localized tacrolimus using a biodegradable nerve wrap or conduit embedded with the drug. Daeschler et al. (2025) and Azapagic et al. (2024) researched this proposed technique on in vivo rat models and were able to generate a release rate of tacrolimus that still allowed statistically significant peripheral nerve regeneration, with a minimal systemic concentration. The nerve conduit designed by Azapagic et al. (2024) released an average of 10 ng/day local tacrolimus, compared to the oral administration of 2–5 mg/day that transplant patients traditionally take. This research has the potential to enhance the recovery of damaged nerves using tacrolimus, but without the original complications (Shokri et al. 2020). However, both research articles are mindful that more extended long‐term studies and human clinical trials will be required before this method can be deemed valid and safe to use.

## 4 Conclusion

Facial transplantation is a modern and innovative surgical procedure with huge potential to transform lives. However, having only become a reality within the last 20 years, it is still considered an experimental practice. With only 50 performed to date, there is a finite amount of literature available. This allowed for thorough and concise collation and analysis of the available literature. However, it also meant that non‐FT‐specific studies were sometimes relied on throughout this comprehensive review.

A successful FT relies on sound knowledge of donor and recipient anatomy. Careful attention to the neurovascular system should optimize aesthetic and functional outcomes. Pre‐operative vascular imaging (CTA or MRA, occasionally followed by DSA) can be used to guide vessel selection. The chosen vessels can then be anastomosed end‐to‐end or end‐to‐side, depending on their route and quality. Continuous open‐loop and interrupted sutures are safe and suitable methods of vascular anastomosis, differing primarily in operative time. Given the time‐intensive nature of FTs, the more efficient open‐loop continuous technique should be favored. Sensory innervation of the graft requires minimal surgical attention due to impressive spontaneous regenerative properties. In contrast, motor innervation can only be restored through coaptation of graft and recipient nerves, with the FN being paramount to secure. Any gap between donor and recipient FN branches should be bridged using a recipient nerve graft, such as the TDN. Microsurgical sutures reinforced by fibrin glue were considered the most suitable method of nerve coaptation. Additional research is needed to clarify the potential of topical tacrolimus in enhancing sensory and motor nerve regeneration in FTs.

When effectively combined, these different pre‐, peri‐, and post‐operative approaches should help construct a graft with maximal neurovascular support, enhancing cosmetic and functional outcomes, as well as recipients' quality of life.

## Ethics Statement

All donations to the University of Leeds have been bequeathed in accordance with the Human Tissue Act 2004. Permission for anatomical examination and imaging was obtained from donors within the bequeathal form prior to the donors' decease.

## Data Availability

The data that support the findings of this study are openly available in PubMed at https://pubmed.ncbi.nlm.nih.gov/?term=Face+Transplant+AND+%28allotransplantation+OR+anatomy+OR+donor+OR+graft+OR+neuro+OR+neurovascular+OR+outcomes+OR+reconstructive+OR+success+OR+surgery+OR+surgical+approaches+OR+transplant+OR+vascularized+composite+allo.
